# City Hospital

**Published:** 1873

**Authors:** 


### CITY HOSPITAL.

The public spirit, the enterprise, the *pluck*, of the people of Atlanta, has almost passed into a proverb. With a faith that knows no doubting, they seize hold of gigantic enterprises with both hands, and by the power of human *will* they compel success, while more timid and conservative communities croak and doubt for a time, and then raise their hands in mute astonishment at the results.

A journeyman tailor, tossing in restless slumber, dreams a dream: From the naked earth there springs up, like Jonah's gourd, in a single night, a vast pile of brick and mortar, which under magical hands takes form and semblance, and straightway there stands a huge hotel facing the Central Depot, and bearing upon its streaming pennons a name and the ever symbolical goose. On the morrow the vapory vision of the night solidifies into determined purpose. The etherial mortar and brick, by the touch of the magician's wand, became fixed and steadfast; the scanty savings of the cutting-board and the workbench swell up into thousands and tens of thousands; the whilom knight of the shears and goose, like the agile mountain *trout*, has leaped at a single bound from his native element into that of a landlord, and dictates terms to boniface.

So likewise out of the coruscation of deflagrating brain, hundreds of thousands in current lucre flow into the magical Gate City. A railroad springs into existence, and the shrill whistle of the locomotive echoes from an unaccustomed quarter. So, too, a huge opera house rises like a toadstool from rank soil, and in turn becomes the capitol of a great State, to shelter her Representative wisdom; and so also rises that stupendous monument of the magical power of faith and will, the H. I. Kimball House. Time would fail us to recount a tithe of the huge enterprises which the pluck of the Atlantese has forced into successful existence—her bed-quilt newspaper enterprises, chartering special railway trains for their transportation; her many large manufacturing establishments, and her magnificent system of public schools.

And now the genius of Atlanta propounds a new scheme, a City Hospital, an asylum for the destitute victims of disease and calamity, and right royally will she execute it. Well does she understand the potency of faith and will, the irresistible power of her pluck. Well does she know the prestige of her past, and the expectation of her future. The public look with confidence for an institution in keeping with her railroads, her public schools, her live newspapers, her opera houses, and her hotels, and they will not look in vain.

There are various points in the location and construction of a hospital which are of the greatest importance in their bearing upon the health and safety of its inmates on the one hand, and the convenience and advantage of the community and the public upon the other. Prominent among the indispensable requisites stands ample provisions for ventilation. This is to be attained by securing, first, a sufficient area of grounds; secondly, by surrounding the establishment, if practicable, with a street upon every side; thirdly, by an ample court in the centre; fourthly by spreading the buildings in such manner as to secure abundant frontage for windows, both upon street and court, and fifthly, ample architectural arrangements by which foul air may escape and pure air may enter.

Closely allied to the necessity for adequate ventilation comes the equal necessity for effective drainage, which, of course, will be amply provided for, and will be duly considered in the selection of a location. Then, too, comes in an ample supply of pure water, which the city authorities are now taking steps to secure in abundance for the city generally, and which the hospital must, of course, have the benefit of. The wards will be lighted with gas, to escape danger of fire, and to escape likewise the deleterious vapors from lamps.

It will not be forgotten that Atlanta is essentially a railroad city, that she is rapidly building up in manufactures, that both now and for the future the City Hospital will be the refuge for the maimed and wounded from these establishments, as well as for the sick. Hence the Hospital will be centrally located, not remote from the Central Depot, that it may be conveniently accessible.

All this the public will expect, and we feel assured their expectation will not be disappointed. B.

